# Cortisol circadian rhythm and jet-lag syndrome: evaluation of salivary cortisol rhythm in a group of eastward travelers

**DOI:** 10.1007/s12020-021-02621-4

**Published:** 2021-02-04

**Authors:** Rosa Maria Paragliola, Andrea Corsello, Eliana Troiani, Pietro Locantore, Giampaolo Papi, Giulia Donnini, Alfredo Pontecorvi, Salvatore Maria Corsello, Cinzia Carrozza

**Affiliations:** 1 Unit of Endocrinology, Università Cattolica del Sacro Cuore, Fondazione Policlinico “Gemelli” IRCCS, Largo A. Gemelli 8, 00168 Rome, Italy; 2 Unit of Chemistry, Biochemistry and Clinical Molecular Biology, Università Cattolica del Sacro Cuore, Fondazione Policlinico “Gemelli” IRCCS, Largo A. Gemelli 8, 00168 Rome, Italy

**Keywords:** Jet-lag, Salivary cortisol, Cortisol circadian rhythm, Eastward travel

## Abstract

**Purpose:**

The activity of the hypothalamus–pituitary–adrenal axis plays a crucial role as an endogenous stress-reactive system. Lifestyle and work often interfere with the endogenous circadian rhythms and can modify the physiological patterns of stress-hormones secretion, including cortisol. We evaluated the cortisol circadian rhythm in the “jet-lag syndrome” that is the most known condition associated with the desynchronization of the circadian rhythm.

**Methods:**

To assess the modifications of cortisol secretion after a long-haul flight, we compared baseline and post-travel salivary cortisol rhythm in a group of 28 healthy eastward travelers (from the U.S.A. or Canada to Italy). The salivary samples were collected about 1 week before the departure at 11 p.m. on day 0 and at 8 a.m., 12 a.m. (midday) and 11 p.m. on day 1 (R0). The same samples were obtained after the landing, the day they flew back home (R1).

**Results:**

Statistical analysis showed a significant difference between R0 and R1 for each sample considered (*p* < 0.005). In particular, the post-travel salivary cortisol levels detected at 11 p.m. both on day 0 and on day 1, were significantly higher than at baseline. Post-travel morning salivary cortisol levels were lower compared with basal rhythm and increased during the morning, reaching the acrophase at 12 a.m.

**Conclusions:**

In eastward travelers, crossing more than five time zones, the cortisol circadian rhythm after the return to the East “remained behind,” being synchronized with the West time. This impaired cortisol secretion can contribute to the pathogenesis of the jet-lag syndrome.

## Introduction

The hormonal response to homeostatic challenge is one of the most important functions of the endocrine system and the activation of the hypothalamus–pituitary–adrenal (HPA) axis represents one of the many endogenous stress-reactive components [1]. In the absence of stimuli, cortisol levels show a peak in the early morning and then decline slowly throughout the day to a nadir in the evening [2, 3]. The circadian rhythm of corticosteroids secretion is regulated by the suprachiasmatic nucleus (SCN) of the hypothalamus. The hypothalamic release of corticotrophin releasing hormone promotes the adrenocorticotropic hormone release from the anterior pituitary gland, which in turn stimulates the adrenal gland to secrete cortisol [4]. Circadian rhythms modulate the synchronization of biological processes with the environment, playing an “internal timekeepers” role mostly based on the exposure to light and dark [5]. The intrinsically photosensitive retinal ganglion cells (ipRGCs) act as circadian phototransducers tuned to discriminate the light from the dark, and play a crucial role in the entrainment of circadian rhythms [6]. The neural impulses are propagated by ipRGCs, via the retinohypothalamic tract directly to the SCN of the hypothalamus. The SCN in turn regulates a set of transcriptional–translational feedback loops that drive the rhythmic circadian expression of the clock components [7, 8]. This transcriptional–translational feedback loop is at the basis of the intrinsic daily circadian rhythm. The sleep schedule also involves the secretion of melatonin (inhibited by the light exposure), which has opposite effects on circadian rhythm entrainment and helps to induce sleep [9].

However, in the modern era, lifestyle and work often are associated with habits interfering with the endogenous circadian rhythms and, consequently, with the physiological patterns of stress-hormones secretion. For example, the exposure to light at night perturbs the circadian rhythm, because light is the major determinant used by the body to discriminate day from night [10]. If light/dark cycles are altered, biological rhythms can become desynchronized.

A well-known condition associated with the desynchronization of the circadian rhythm is the so-called “jet-lag syndrome,” associated with long-haul flights across several time zones. The cause of jet lag is the persistence of the “body clock” function in the day–night rhythm of the place of departure. This syndrome causes the lack of alertness, poor sleep, irritability, stress, impaired performance in athletes, and depressed mood [11]. Considering the role of the HPA axis in the regulation of the stress–response, cortisol can be considered a reliable marker of jet-lag syndrome and the disruption of glucocorticoid rhythmicity is probably implicated in the above mentioned physical alterations.

In this study, we evaluated the cortisol circadian rhythm, by salivary cortisol measurement, in a group of healthy volunteers during intercontinental travel. The main goal was to compare the curve of cortisol secretion during the “jet-lag syndrome” with a baseline profile obtained before the departure. We used salivary cortisol because it is an easy and noninvasive method to check for cortisol rhythm in the clinical practice, which reflects the serum-free cortisol concentration [12].

## Materials and methods

We compared baseline and post-travel salivary cortisol rhythms in a group of healthy West-to-East flyers. We included 28 healthy volunteers who traveled by a direct flight to the U.S.A. or Canada between March and August 2019. Informed consent has been obtained by the participants. We enrolled only subjects who were not on therapy with steroids, steroidogenesis inhibitors or drugs interfering with cortisol metabolism and who did not perform night shifts. Even if salivary cortisol measurement is not affected by interference from estrogens, we did not include neither pregnant women nor women on therapy with oral contraceptives. No subject had clinical history of pituitary or adrenal disease.

The salivary samples were collected by the participants as follows:

– About 1 week before the departure from Italy, at 11 p.m. on day 0 and at 8 a.m., 12 a.m. (midday) and 11 p.m. on day 1 (“basal rhythm,” R0);
– After the landing when they flew back to Italy (day 0) at 11 p.m. on day 0 and at 8 a.m., 12 a.m. and 11 p.m. on day 1 (“fly-back rhythm,” R1).

The volunteers collected the saliva using a commercially available device (Salivette^®^, Sarstedt). We provided each patient with instructions about how to collect salivary samples. In particular, the use of licorice or cigarettes before sample collection was forbidden as well as sample collection in cases of gums or oral mucosa bleeding. The Salivette^®^ devices have been stored at 5 °C in volunteers’ domestic refrigerators. Then, each device has been carried to Policlinico Gemelli Biochemistry and Clinical Chemistry laboratory within 48 h from the saliva collection.

Salivary samples were centrifuged at 4 °C for 10 min and stored at −80° until assayed. Salivary cortisol was assayed by an electrochemiluminescence immunoassay Roche^®^ on Elecsys Cobas E411. The analytical and functional sensitivity of the method was 0.6 and 1.9 nmol/L, respectively. Intra- and interassay coefficient of variation was <10%. The diagnostic accuracy of this method has been evaluated in a previous paper in a group of Cushing’s syndrome patients by ROC curve analysis. A cutoff value of 8.3 nmol/L has been found, leading to 100% sensitivity and 98% specificity. The diagnostic accuracy was 97% [12]. Normal salivary cortisol levels have been reported in Table 1. We obtained our own reference range for salivary cortisol levels at 8 and 12 a.m. from 40 healthy volunteers. Late-night normal salivary cortisol levels have been previously obtained in our institution, based on midnight salivary cortisol in a group on healthy subjects [12]. Statistical analysis has been performed by using Student *t*-test for paired samples (Microsoft Excel 2019^®^). Data have been expressed as mean ± SD and *p* value < 0.05 has been considered significant.

**Table 1:** Salivary cortisol normal levels in healthy subjects

	Salivary cortisol normal levels (nmol/L)
Morning (6:00–10:00 a.m.)	18.8 ± 3.6
Afternoon (4:00–8:00 p.m.)	4.9 ± 1.89
Late-night (11:00–12:00 p.m.)	<3.03

Furthermore, we asked the volunteers to report the clinical symptoms occurring during the day 1 after the fly-back home. We administered to each subject a modified version of the “Liverpool jet-lag questionnaire” [13], a symptom-based questionnaire composed by six items (Table 2). We divided the patients in three subgroups for each item on the basis of the symptoms reported as shown in Table 2. The results of the Liverpool questionnaire have been correlated with all R1 salivary cortisol samples. Statistical analysis has been performed with the Mann–Whitney test for non-paired data.

**Table 2:** The Liverpool jet-lag questionnaire (modified)

Items and scores	No. of subjects
**1. Jet lag: how much jet lag do you have?**	
Insignificant (from 0 to 3)	6
Mild jet-lag (from 4 to 7)	16
Very bad (from 8 to 10)	6
**2. Last night’s sleep. When compared with normal:**	
a. How easily did you get to sleep?	
Less (from −5 to −1)	13
Normal 0	5
More (from +1 to +5)	10
b. What time did you get to sleep?	
Earlier (from −5 to −1)	6
Normal 0	6
Later (from +1 to +5)	16
c. How well did you sleep?	
More waking episodes (from −5 to −1)	20
Normal 0	2
Fewer waking episodes (from +1 to +5)	6
d. What was you waking time?	
Earlier (from −5 to −1)	11
Normal 0	3
Later (from +1 to +5)	14
e. How alert did you feel 30 min after rising?	
Less (from −5 to −1)	26
Normal 0	2
More (from +1 to +5)	0
**3. Fatigue: in general, compared to normal how tired do you feel at the moment?**	
More (from −5 to −1)	0
Normal 0	0
Less (from +1 to +5)	28
**4. Meals. Compare with normal:**	
a. How hungry did you feel before your meal?	
Less (from −5 to −1)	9
Normal 0	7
More (from +1 to +5)	12
b. How palatable (appetising) was the meal?	
Less (from −5 to −1)	9
Normal 0	11
More (from +1 to +5)	8
c. After your meal, how do you now feel?	
Still hungry (from −5 to −1)	7
Satisfied 0	9
Bloated (from +1 to +5)	12
5. Mental performance and mood. Compared with normal:	
a. How well have you been able to concentrate?	
Worse (from −5 to −1)	21
Normal 0	5
Better (from +1 to +5)	2
b. How motivated do you feel?	
Less (from −5 to −1)	11
Normal 0	9
More (from +1 to +5)	8
c. How irritable do you feel?	
Less (from −5 to −1)	6
Normal 0	7
More (from +1 to +5)	15
**6. Bowel activity today. Compared with normal:**	
a. How frequent have your bowel motions been?	
Less (from −5 to −1)	16
Normal 0	8
More (from +1 to +5)	4
b. How has the consistency been?	
Harder (from −5 to −1)	17
Normal 0	7
Looser (from +1 to +5)	4

The subjects have filled in the questionnaire based on a score. For the item *n*.1 the score is from 0 to 10; for the other items, the score is from −5 to +5. The subjects have been classified in three groups according to the response for each item (in the round brackets the range of the score associated to each group has been reported)

## Results

Twelve females and sixteen males (mean age 37 ± 14 years) were included in the study. Subjects flew back from West-to-East (from the U.S.A. or Canada to Rome). The mean permanence in the U.S.A. or Canada was 13.6 ± 2.4 days (between 10 and 16 days). Traveling from West-to-East, they crossed between 5 and 8 time zones (mean 6.32 ± 0.96). The time between the fly-back landing in Rome and the first R1 sample collection (11 p.m. of day 0) was 13.12 ± 1.23 h. All subjects landed in Rome between 7.30 a.m. and 12 a.m.

We obtained 224 samples [112 included in the group “basal rhythm” (R0) and 112 included in the group “fly-back rhythm” (R1)]. Salivary cortisol levels have been reported in Table 3 (mean ± SD). Each sample of the two groups (R0 and R1) has been named and grouped based on the time of collection: sample 1 (day 0, 11 p.m.), sample 2 (day 1, 8 a.m.), sample 3 (day 1, 12 a.m.), and sample 4 (day 1, 11 p.m.).

**Table 3:** Basal rhythm (R0) and fly-back rhythm (R1) salivary cortisol levels (data expressed as mean ± SD)

No. of sample	Time of sample	Basal salivary cortisol rhythm (R0) (nmol/L)	Fly-back cortisol rhythm (R1) (nmol/L)	*p*
1	11 p.m. day 0	1.38 ± 0.00	4.82 ± 6.73	**0.0099**
2	8 a.m. day 1	17.25 ± 3.42	4.11 ± 3.93	**<0.000**
3	12 a.m. day 1	3.05 ± 1.07	7.37 ± 6.80	**0.0016**
4	11 p.m. day 1	1.46 ± 0.22	3.78 ± 4.35	**0.0079**

Student *t*-test comparing R0 and R1 showed significant differences for each time of sample (reported as sample ns. 1, 2, 3, and 4) (significance for *p* < 0.05)

In bold, *p*-values < 0.05.

The “basal rhythm” (R0) resulted normal in every single volunteer, confirming the absence of alteration of the HPA axis.

**Fig. 1:**
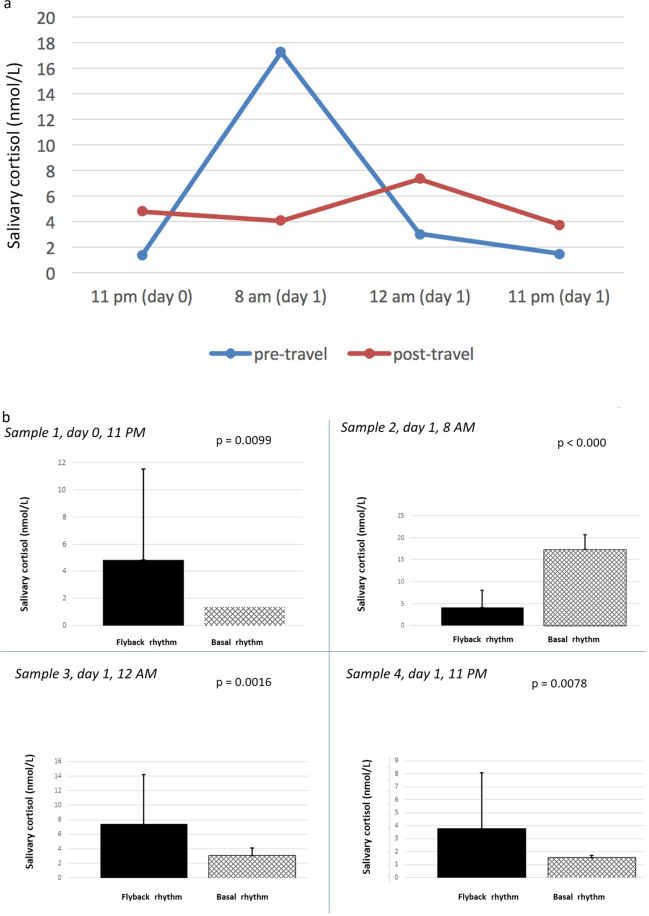
Comparison between mean basal and fly-back salivary cortisol levels overall (**a**) and for each sample (**b**). Samples 1 and 4 (11 p.m. on days 0 and 1, respectively) showed fly-back salivary cortisol levels significantly higher than at baseline. On the contrary, post-travel morning salivary cortisol levels were lower compared with basal rhythm (sample 2). An increase of salivary cortisol levels occurred during the morning, reaching the acrophase at 12 a.m. (sample 3)

Statistical analysis showed a significant difference between R0 and R1 for each sample considered (*p* < 0.05, Table 3). In particular, the post-travel salivary cortisol levels detected at 11 p.m. both on day 0 and on day 1, were significantly higher than at baseline. On the contrary, post-travel morning salivary cortisol levels were lower compared with basal rhythm and increased during the morning, reaching the acrophase at 12 a.m., as shown in Fig. 1. No significant correlation in 11 p.m. post-travel salivary cortisol between the day 0 and the day 1 (R1, samples 1 and 4) was observed.

About clinical symptoms, 6 of 28 subjects had a “very-bad jet-lag,” 14 subjects had a mild jet-lag while 6 subjects had an insignificant jet-lag. All subjects reported fatigue. Two of 28 subjects did not report any other symptoms. The results of all the items of Liverpool questionnaire have been reported in Table 2. Statistical analysis did not show any correlation between jet-lag occurrence or clinical symptoms and fly-back salivary cortisol rhythm.

## Discussion

In this study we evaluated the pattern of cortisol secretion after an intercontinental West-to-East fly. In our results, we observed that the intercontinental travel significantly influenced the circadian cortisol rhythm, causing an inversion of the physiological pattern of cortisol secretion. In particular, post-travel late-night salivary cortisol was higher when compared with the physiologic undetectable midnight salivary cortisol of the basal rhythm. Interestingly enough, this elevation in late-night salivary cortisol persisted at 24 h (i.e., 11 p.m. of day 1). On the contrary, the nadir in fly-back cortisol rhythm was observed at 8 a.m. of day 1 and was followed by an increase in salivary cortisol levels that reached their acrophase at 12 a.m. Therefore, we can assume that, after a West-to-East fly, the set-point of cortisol secretion remains still synchronized on the West time zone for at least 36 h from the return to East. This process represents an extreme loss of synchronization between internal circadian rhythm and external time, which in general causes the jet-lag syndrome. However, in our study, we did not find a significant correlation between clinical symptoms and after travel salivary cortisol levels.

The first definition of jet lag appeared in in 1966 on Los Angeles Times: “If you’re going to be a member of the Jet Set and fly off to Katmandu for coffee with King Mahendra, you can count on contracting Jet Lag, a debility not unakin to a hangover. Jet Lag derives from the simple fact that jets travel so fast they leave your body rhythms behind” (Horace Sutton) [14]. In the same period, a study of the Federal Aviation Agency evaluated the conditions that produce pilot fatigue, underlining the effects, for the intercontinental-air-carrier crew, of the disruption of the physiological day–night cycling occurring for the rapid translocation through many time zones [8, 15–17].

Interestingly, the direction of travel can affect the occurrence and the severity of the jet-lag syndrome with symptoms and sleep disturbances being worse after eastward (West-to-East) flights than after westward (East-to-West) flights. Indeed, traveling in an eastward direction, the length of the day is shortened, and the circadian system must become shorter to re-establish a normal rhythm, that is more difficult to achieve than to adjust a “longer” rhythm as it occurs in westward travels [16].

Contrary to the so-called “travel fatigue” (anxiety about the journey, changes to an individual’s daily routine, and dehydration due to time spent in the aircraft), which in general is shorter, lasts for only a day and occurs in all long travels, included flies North-to-South and vice-versa [17], the jet-lag syndrome is more prolonged, more complex and occurs only in the travel crossing different time zones (transmeridian travels).

The adrenal circadian clock, through the control of glucocorticoid rhythms, seems to represent a major regulator of re-entrainment to jet lag [8, 18].

Based on these knowledges, we tried to test this hypothesis testing directly the cortisol rhythm in human subjects. We used devices for salivary cortisol collection, which represents a simple, stress-free, and reliable tool for the investigation of HPA axis activity [19].

Other authors investigated the effect of jet-lag evaluating salivary cortisol. Bullock and coauthors evaluated a small group of athletes (five subjects) who had an eastward fly from Australia to Canada. Awakening salivary cortisol decreased by 67% immediately after the travel, compared with baseline levels. Interestingly, on day 1 after the flight the salivary cortisol concentrations were similar among all athletes, while during the first 4 days after the flight, salivary cortisol concentrations varied between the subjects, suggesting an inter-individual variability of the rates of re-synchronization [20].

Another study from the U.S.A. in 2010, including 764 middle-aged men, showed that eastward travels were associated with a steeper salivary cortisol awakening response and lower peak levels of salivary cortisol the next morning. Westward travels showed lower peak levels of cortisol the next morning. However, despite the size of the sample, the participants were only men from the Vietnam War. Even if the authors specified that post-traumatic stress disturbance did not represent a bias for the results, they excluded the women and the younger population. Furthermore, the study investigated relatively short-distance travels (three or fewer time zones) [21].

In our study, we considered a population including both sexes and crossing between 5 and 8 time zones. The sample was homogeneous regarding the days of stay in western countries and the hours between the landing and the collection of the first sample. Furthermore, we evaluated only eastward travelers. All participants reported fatigue, while in the majority of cases other symptoms of jet lag occurred, and in particular sleep disturbances even in absence of significant correlation with salivary cortisol rhythm.

The main limitations of our study are the small size of our sample and the short duration of post-travel salivary collection, which did not allow us to check for how long the cortisol secretion rhythm disruption persisted. However, considering the easiness in the collection samples by the salivary cortisol devices, it will be possible to extend the study to a wide number of participants for a longer time.

## Conclusion

In our study, we confirmed that cortisol circadian rhythm is disrupted after a travel crossing more than five time zones. This clinical study reinforces the role of cortisol secretion in the pathogenesis of the jet-lag syndrome. In particular, we focused our evaluation on the eastward travelers and confirmed, according to other findings reported in literature, that the cortisol circadian rhythm after the return to the East, “remained behind” and was synchronized with the West time.

## Data Availability

The datasets generated during and/or analysed during the current study are available from the corresponding author on reasonable request.

## References

[1] Herman JP, McKlveen JM, Ghosal S, Kopp B, Wulsin A, Makinson R, Scheimann J, Myers B (2016). Regulation of the hypothalamic-pituitary-adrenocortical stress response. Compr. Physiol..

[2] Horrocks PM, Jones AF, Ratcliffe WA, Holder G, White A, Holder R, Ratcliffe JG, London DR (1990). Patterns of ACTH and cortisol pulsatility over twenty-four hours in normal males and females. Clin. Endocrinol..

[3] Krieger DT, Allen W, Rizzo F, Krieger HP (1971). Characterization of the normal temporal pattern of plasma corticosteroid levels. J. Clin. Endocrinol. Metab..

[4] Chan S, Debono M (2010). Replication of cortisol circadian rhythm: new advances in hydrocortisone replacement therapy. Ther. Adv. Endocrinol. Metab..

[5] Walker WH, Walton JC, DeVries AC, Nelson RJ (2020). Circadian rhythm disruption and mental health. Transl. Psychiatry.

[6] Kofuji P, Mure LS, Massman LJ, Purrier N, Panda S, Engeland WC (2016). Intrinsically photosensitive retinal ganglion cells (ipRGCs) are necessary for light entrainment of peripheral clocks. PLoS ONE.

[7] Buhr ED, Takahashi JS (2013). Molecular components of the mammalian circadian clock. Handb. Exp. Pharm..

[8] Kiessling S, Eichele G, Oster H (2010). Adrenal glucocorticoids have a key role in circadian resynchronization in a mouse model of jet lag. J. Clin. Invest..

[9] Atkinson G, Drust B, Reilly T, Waterhouse J (2003). The relevance of melatonin to sports medicine and science. Sports Med..

[10] Potter GD, Skene DJ, Arendt J, Cade JE, Grant PJ, Hardie LJ (2016). Circadian rhythm and sleep disruption: causes, metabolic consequences, and countermeasures. Endocr. Rev..

[11] Herxheimer A, Waterhouse J (2003). The prevention and treatment of jet lag. BMJ.

[12] Carrozza C, Corsello SM, Paragliola RM, Ingraudo F, Palumbo S, Locantore P, Sferrazza A, Pontecorvi A, Zuppi C (2010). Clinical accuracy of midnight salivary cortisol measured by automated electrochemiluminescence immunoassay method in Cushing’s syndrome. Ann. Clin. Biochem..

[13] Waterhouse J, Reilly T, Atkinson G, Edwards B (2007). Jet lag: trends and coping strategies. Lancet.

[14] Arendt J (2018). Approaches to the pharmacological management of jet lag. Drugs.

[15] G.T. Hauty, T. Adams, Aerospace Medicine Technical Reports. in *Pilot Fatigue: Intercontinental Jet Fight* (Federal Aviation Agency—Office of Aviation Medicine, 1965). Report NO: DOT/FAA/AM-65/16. https://www.faa.gov/data_research/research/med_humanfacs/oamtechreports/1960s/media/AM65-16.pdf

[16] Lee A, Galvez JC (2012). Jet lag in athletes. Sports Health.

[17] Waterhouse J, Reilly T, Edwards B (2004). The stress of travel. J. Sports Sci..

[18] Balsalobre A, Brown SA, Marcacci L, Tronche F, Kellendonk C, Reichardt HM, Schutz G, Schibler U (2000). Resetting of circadian time in peripheral tissues by glucocorticoid signaling. Science.

[19] Kirschbaum C, Hellhammer DH (1989). Salivary cortisol in psychobiological research: an overview. Neuropsychobiology.

[20] Bullock N, Martin DT, Ross A, Rosemond D, Marino FE (2007). Effect of long haul travel on maximal sprint performance and diurnal variations in elite skeleton athletes. Br. J. Sports Med..

[21] Doane LD, Kremen WS, Eaves LJ, Eisen SA, Hauger R, Hellhammer D, Levine S, Lupien S, Lyons MJ, Mendoza S, Prom-Wormley E, Xian H, York TP, Franz CE, Jacobson KC (2010). Associations between jet lag and cortisol diurnal rhythms after domestic travel. Health Psychol..

